# OxyR-activated expression of Dps is important for *Vibrio cholerae* oxidative stress resistance and pathogenesis

**DOI:** 10.1371/journal.pone.0171201

**Published:** 2017-02-02

**Authors:** Xiaoyun Xia, Jessie Larios-Valencia, Zhi Liu, Fu Xiang, Biao Kan, Hui Wang, Jun Zhu

**Affiliations:** 1 College of Life Sciences, Nanjing Agricultural University, Nanjing, China; 2 State Key Laboratory for Infectious Disease Prevention and Control, National Institute for Communicable Disease Control and Prevention, Chinese Center for Disease Control and Prevention, Beijing, China; 3 Department of Microbiology, University of Pennsylvania Perelman School of Medicine, Philadelphia, Pennsylvania, United States of America; 4 Department of Biotechnology, Huazhong University of Science and Technology, Wuhan, China; 5 College of Life Sciences, Huanggang Normal University, Huanggang, China; 6 Collaborative Innovation Center for Diagnosis and Treatment of Infectious Diseases, Hangzhou, China; University of Massachusetts Medical School, UNITED STATES

## Abstract

*Vibrio cholerae* is the causative agent of cholera, a dehydrating diarrheal disease. This Gram-negative pathogen is able to modulate its gene expression in order to combat stresses encountered in both aquatic and host environments, including stress posed by reactive oxygen species (ROS). In order to further the understanding of *V*. *cholerae’s* transcriptional response to ROS, we performed an RNA sequencing analysis to determine the transcriptional profile of *V*. *cholerae* when exposed to hydrogen hydroperoxide. Of 135 differentially expressed genes, VC0139 was amongst the genes with the largest induction. VC0139 encodes a protein homologous to the DPS (DNA-binding protein from starved cells) protein family, which are widely conserved and are implicated in ROS resistance in other bacteria. Using a promoter reporter assay, we show that during exponential growth, *dps* is induced by H_2_O_2_ in a manner dependent on the ROS-sensing transcriptional regulator, OxyR. Upon entry into stationary phase, the major stationary phase regulator RpoS is required to transcribe *dps*. Deletion of *dps* impaired *V*. *cholerae* resistance to both inorganic and organic hydroperoxides. Furthermore, we show that Dps is involved in resistance to multiple environmental stresses. Finally, we found that Dps is important for *V*. *cholerae* adult mouse colonization, but becomes dispensable in the presence of antioxidants. Taken together, our results suggest that Dps plays vital roles in both *V*. *cholerae* stress resistance and pathogenesis.

## Introduction

The human pathogen *Vibrio cholerae*, a motile gram-negative bacterium, is the causative agent of the waterborne disease, cholera [1, 2] that is still a major threat to public health in developing countries [3]. *V*. *cholerae* survives in various environments by sensing and responding to environmental cues. Its pathogenesis is dependent on the oral-fecal route, where it enters the human gastrointestinal tract through oral ingestion and propagates its own release into the environment through toxin production that causes choleric diarrhea [4]. Within a human host, *V*. *cholerae* senses signals such as changing oxygen tension and the presence of bile salts and bicarbonate, enabling the activation of a regulatory cascade leading to virulence gene expression [5–8]. *V*. *cholerae* also encounters oxidative stress during the later stages of infection [9, 10] as well as in the aquatic environment [11]. In *V*. *cholerae*, the ROS-sensing activator OxyR is important for resistance to hydrogen peroxide [12], while OhrR, a regulator of the organic hydroperoxide resistance gene *ohrA*, regulates *V*. *cholerae* resistance to organic hydroperoxides [13]. Quorum sensing systems [14] and the virulence regulator AphB also play important roles in oxidative stress response [15].

Oxidative stress response regulation in bacteria has been extensively studied [16]. Many bacteria have evolved sophisticated regulatory systems to overcome ROS that are acutely toxic to bacterial cells. For example, during oxidative stress, *Escherichia coli* utilizes OxyR and SoxRS to sense ROS signals and subsequently coordinate the expression of a set of genes encoding ROS scavenging enzymes, such as catalases and peroxidases [17]. In addition, Dps (the DNA-binding protein from starved cells), a non-specific DNA-binding protein, has been known to be implicated in *E*. *coli* ROS resistance [18, 19]. Dps is the most abundant protein in stationary phase cells, and has been shown to be regulated by OxyR during exponential phase and RpoS during stationary phase [20–22]. The non-specific DNA binding of Dps protects DNA against ROS through the physical association with DNA and the ability to nullify the toxic combination of Fe (II) and H_2_O_2_ [23]. In addition to playing a role in oxidative stress resistance [24–26], Dps is also involved in *E*. *coli* resistance to acid stress [27], iron and copper toxicity [25, 26, 28]. Homologues of Dps are widely distributed throughout bacteria and are important for ROS resistance and other physiological functions such as pathogenesis [29–31]. In this study, using RNA sequencing and transcriptional reporters, we found that *V*. *cholerae dps* expression is induced by hydrogen peroxide in an OxyR-dependent manner. Deletion analysis indicates that Dps is important for *V*. *cholerae* oxidative stress resistance and pathogenesis.

## Materials and methods

### Ethics statement

These studies were limited to the use of mice only. The protocol was approved by the Ethical Committee of Animal Experiments of Nanjing Agricultural University (Permit Number: SYXK (su) 2011–0036). All efforts were made to minimize animal suffering and the number of animals to be used. After infection, mice were monitored until awake and were monitored for signs of distress throughout the duration of experiments. Moribund animals, or animals that appeared to be experiencing pain or suffering, were sacrificed at earlier time points. Upon termination of experiments, the adult mice were euthanized by CO_2_ inhalation followed by decapitation.

### Strains, plasmids and culture conditions

All strains used in this study were derived from *V*. *cholerae* El Tor C6706 [32]. In-frame deletions of *dps* and *rpoS* mutants were constructed by cloning the regions flanking the gene of interest into suicide vector pWM91 containing a *sacB* counter-select marker [33]. Double-crossover recombinant mutants were selected using sucrose plates. The construction of *oxyR* mutants is described in [12]. The P_*dps*_-*lux* transcriptional fusion reporter was constructed by cloning *dps* promoter sequences into pBBR-lux which contains a promoterless *luxCDABE* reporter [34]. The P_*tcpA*_-*lux* construct is described in [35]. The *dps* overexpression plasmid was constructed by cloning the PCR-amplified coding regions into pBAD24 [36] and the construction of the *oxyR* overexpression plasmid is described in [12]. Strains were propagated in LB containing appropriate antibiotics at 37°C, unless otherwise noted.

### RNA sequencing

Wild type *V*. *cholerae* were inoculated into AKI medium [37] and incubated without shaking for 4 hrs at 37°C. One set of cultures were then exposed to 0.5 mM H_2_O_2_ for 30 min. RNA was then purified using TRIzol® (ThermoFisher Sci) and RNeasy purification kits (Qiagen). RNA sequencing was performed by PrimBio Research Institute LLC (Exton, PA, USA). Ribo-Zero rRNA Removal Kit (Bacteria) (Illumina) was used for rRNA removal. Subsequently, the rRNA- depleted RNA was used to construct a cDNA library. cDNA libraries were then constructed using the Ion Total RNA-Seq Kit (Life Technologies). The purified cDNA libraries were then amplified by PCR using Platinum PCR Super-Mix High Fidelity and Ion Xpress RNA Barcode reverse and forward primers. Approximately 10 pM of pooled barcoded libraries were then used for templating using Life Technologies Ion PI IC 200 Kit. Samples were then loaded on Ion P1 chips for Ion Torrent RNA-Seq. Following proton run, the raw sequences were aligned to the *V*. *cholerae* N16961 genome. Aligned BAM files were used for further analysis. BAM files, separated by the specific barcodes, were uploaded to the Strand NGS software (San Francisco, CA). Quality control was assessed by the Strand NGS program, which determined the pre- and post-alignment quality of the reads for each sample. The aligned reads were then filtered based on alignment score, match count, mapping quality, and average base quality. After filtering, the aligned reads were normalized and quantified using the Deseq algorithm by Strand NGS. The standard t-test was used to determine significant differentially expressed genes based on two biological replicates of each condition. Sequencing data for RNA-seq experiments are accessible at SRP095162 in the Sequence Read Archive (SRA).

### Measuring *dps* expression using transcriptional reporters

Overnight cultures of wild type, *ΔoxyR*, *ΔoxyR* (pBAD-*oxyR*), and *ΔrpoS*, all containing P_*dps*_-*luxCDABE* transcriptional fusion plasmids were diluted into fresh LB containing appropriate antibiotics and shaken at 37°C until early-log/mid-log/late-log or stationary phase. When indicated, cultures were exposed to H_2_O_2_ and were incubated for 1 hr. When appropriate, culture medium was supplemented with 0.1% arabinose. Luminescence was then measured and normalized to OD_600._ Three independent experiments were performed.

### ROS resistance assays

Overnight cultures of wild type, Δ*dps*, and Δ*dps* (pBAD-*dps*) were diluted 1:1000 into LB containing 0.1% arabinose without or with 250 μM H_2_O_2_ or 80 μM cumene hydroperoxide and incubated aerobically at 37°C. OD_600_ was measured at the indicated time points. Three independent experiments were performed.

### In vitro stress assays

Mid-log, stationary-phase, and starved cultures (mid-log cultures starved in artificial sea water (ASW) at 22°C for 2 days) of wild type and *dps* mutants were exposed to the following stress conditions: 2 mM H_2_O_2_ or 3 mM cumene hydroperxide (CHP) exposure for 15’, 10 mM FeSO_4_ challenge for 15’, and acid shock (pH 4.5) for 30’. Survival rate was then determined by plating samples on LB plates after serial dilution and percent survival was calculated by comparing with unexposed cells. For high osmolality stress assay, different growth-staged cultures of wild type and *dps* mutant cells were incubated in 1 M NaCl at 37°C for 24 hrs. Percentages of surviving cells were calculated by comparing with the number of cells surviving in 0.8% NaCl /LB (pH 7). Three independent experiments were performed.

### VBNC assays

*V*. *cholerae* viable but not culturable (VBNC) assays were performed as described in [38]. Briefly, late-log LB cultures of wild type and Δ*dps* were washed and diluted in ASW to a final concentration of 10^8^ CFU/ml. The cell suspensions in artificial sea water were then incubated at 4°C for 70 days. At the indicated time, the number of culturable cells was determined by plating the cell suspension on tryptic soy agar plates supplemented with 0.1% sodium pyruvate. To determine the number of viable cells, samples were treated with propidium monoazide (PMA), which is a DNA-binding PCR inhibitor that selectively crosses compromised cell membranes. After PMA treatment, DNA was isolated from samples and quantitative real time PCR assays were performed by using primers for VC1376. Three independent experiments were performed.

### *In vitro* assays for *tcpA* expression and TCP pilin production

Overnight cultures of wild type and Δ*dps* containing P_*tcpA*_-*luxCDABE* transcriptional fusion plasmids were inoculated 1:10000 into AKI medium [37] and incubated without shaking at 37°C for 4 hrs, followed by shaking at 37°C for an additional 3 hrs. Luminescence was then measured at the indicated time points and normalized to OD_600_. At the final time point, 10^9^ cells were subjected to sodium dodecyl sulfate-polyacrylamide gel electrophoresis (SDS-PAGE) and immunoblotting using anti-TcpA antiserum. Three independent experiments were performed. Representative data are shown.

### *In vivo* competition colonization assay

The infant mouse model was used as previously prescribed [39]. Briefly, overnight cultures of wild type (*lacZ*^+^) and Δ*dps* (*lacZ*^-^) were mixed in a 1:1 ratio and approximately 10^5^ *V*. *cholerae* cells were intragastrically inoculated into 5-day-old CD-1 suckling mice. After an 18hr infection period, the mice were sacrificed. Small intestines were homogenized and the ratio of mutants to wild type was determined by plating on LB agar containing 5-bromo-4-chloro-3-indolyl-β-D-galacto-pyranoside (X-Gal). Each experiment consisted of a sample size of 5 mice.

The streptomycin-treated adult mouse model was used as previously described [13]. Five-week-old CD-1 mice were provided drinking water with or without the antioxidant, N-acetyl cysteine (NAC) [1% (wt/vol)] for one week. 0.5% (wt/vol) streptomycin and 0.4% aspartame were then added to the drinking water for the remainder of the experiment. One day after streptomycin treatment, approximately 10^8^ wild type and Δ*dps* cells were mixed in a 1:1 ratio and intragastrically administered to each mouse. Fecal pellets were collected at the indicated time points, resuspended in LB, serially diluted, and plated on plates containing X-gal. The competitive index was calculated as the ratio of mutant to wild type colonies normalized to the input ratio. Each experiment consisted of a sample size of 5 mice.

## Results and discussion

### Global transcriptomic responses of *V*. *cholerae* to hydrogen peroxide

**Fig 1:**
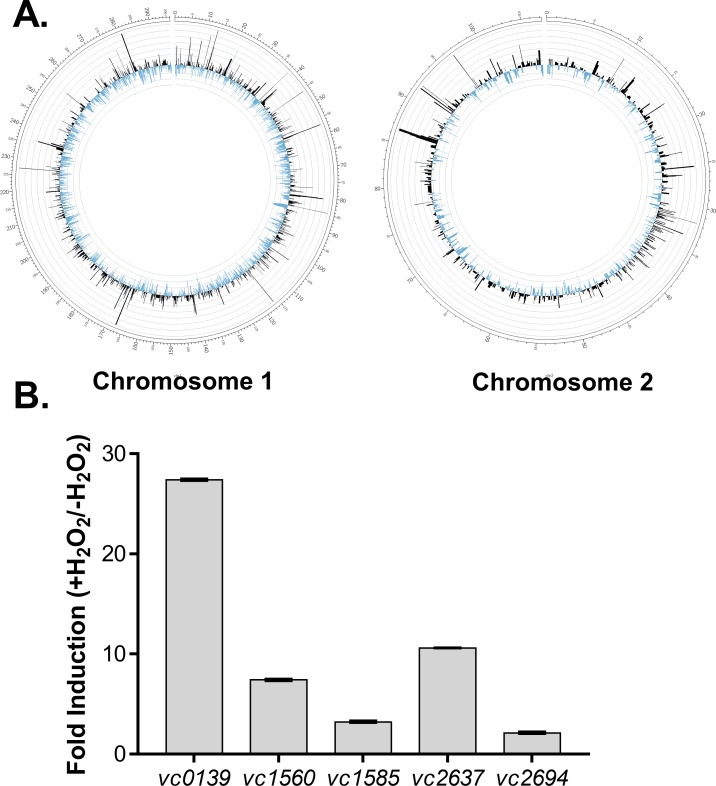
Global transcriptional profiling of *V*. *cholerae* in response to hydrogen peroxide. Wild type *V*. *cholerae* were grown in AKI medium [37] without shaking for 4 hrs. One set of cultures were then exposed to 0.5 mM H_2_O_2_ for 30 min. RNA was extracted and then subjected to RNA sequencing analysis. **A.** Chromosomal location of differentially expressed genes. The map was constructed using the Circos program [43]. Fold-changes of genes upregulated by H_2_O_2_ are labeled in black and downregulated labeled in blue. **B.** Highly induced *V*. *cholerae* genes by H_2_O_2_.

In order to study how *V*. *cholerae* manipulate their genetic reservoirs to resist oxidative stress, we analyzed the transcriptome of *V*. *cholerae* grown in the presence and absence of hydrogen peroxide using RNA-seq. We grew *V*. *cholerae* in AKI medium [37], in which virulence genes are highly induced, until mid-log phase. Cultures were then exposed to 0.5 mM H_2_O_2_ and further incubated at 37°C for 30 min. Total RNA was then harvested and subjected to subsequent RNA-seq. Read mapping against the *V*. *cholerae* N16961 genome was performed and allowed for the identification of differentially expressed genes. The analysis identified the expression of 3689 coding DNA sequence (CDS) tags. Biological replicates were tightly clustered, indicating consistency between replicates. As shown in S1 Table, we identified a total of 135 genes that displayed at least 2-fold differential expression upon H_2_O_2_ exposure. These genes are scattered along the two chromosomes of the *V*. *cholerae* genome (Fig 1A). Among those differentially expressed genes, expression of 99 genes was repressed in the presence of H_2_O_2_. Many of the downregulated genes are related to primary metabolism and cellular transport systems, suggesting that hydrogen peroxide attenuates cellular metabolism and transport through the cell membrane. Similar phenotypes were also observed in *Pseudomonas aeruginosa* when exposed to sublethal doses of H_2_O_2_ [40]. Interestingly, transcription of the key virulence regulator ToxR, which is essential for *V*. *cholerae* pathogenesis [41], was repressed 2.1-fold by H_2_O_2_, indicative of a relationship between ROS resistance and *V*. *cholerae* pathogenesis. How oxidative stress influences virulence is subject to another study.

Many of the genes induced by H_2_O_2_, are known to be involved in cellular protective mechanisms (Fig 1B). For example, expression of peroxiredoxin PrxA (VC2637) showed an over 10-fold increase in response to hydrogen peroxide. PrxA in *V*. *cholerae* is important for ROS resistance and is induced by H_2_O_2_ [12, 42]. Both catalase genes, VC1560 (*katG*) and VC1585 (*katB*) were also induced. We previously showed [12] that both of these catalases are critical for *V*. *cholerae* survival upon exposure to H_2_O_2_. Amongst these strongly induced genes was VC0139, induced over 25-fold, which putatively encodes a Dps family protein. As Dps family proteins have been shown to be involved in ROS resistance in many other bacteria, we chose to further investigate Dps.

### *dps* expression is controlled by OxyR and RpoS at different growth phases

**Fig 2:**
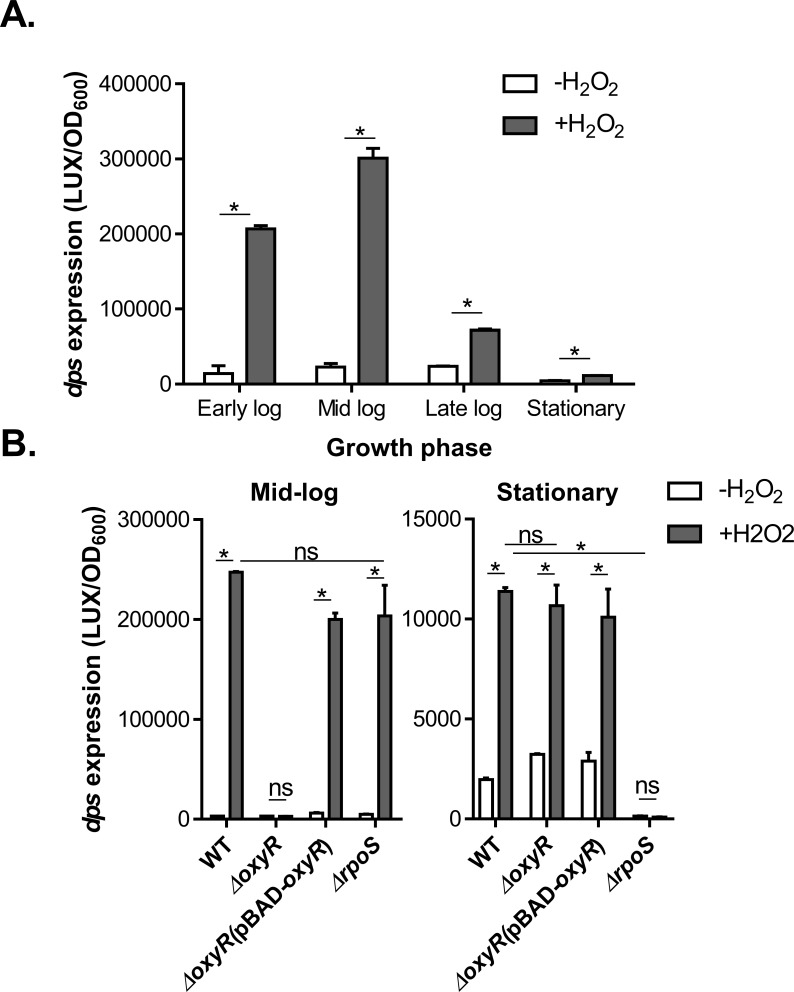
The effects of H_2_O_2_ and OxyR on *dps* transcription. **A.** *dps* expression at distinct growth stages. Wild type *V*. *cholerae* containing P_*dp*s_-*luxCDABE* reporter plasmids were grown to the indicated time points, 250 μM H_2_O_2_ were added and incubated for an additional hour. Luminescence was measured and normalized to OD_600_. Results are means and standard deviations of three independent experiments. **B.** The effects of OxyR and RpoS on *dps* expression during different growth phases. Wild type, *ΔoxyR*, *ΔoxyR* (pBAD-*oxyR*), *ΔrpoS* containing P_*dp*s_-*luxCDABE* reporter plasmids were grown to the indicated growth phase, 100 μM (early-log) or 500 μM H_2_O_2_ (mid-log and stationary) were added and incubated for an additional hour. Luminescence was measured and normalized to OD_600_. When indicated, 0.1% arabinose was added in the medium to induce the P_BAD_ promoter. Results are means and s.d. of three independent experiments. *: Student t-test, P<0.05; ns: no significance.

To verify RNA-seq results, we constructed a P_*dps*_*-luxCDABE* transcriptional fusion plasmid to monitor *dps* expression. We found that in the absence of H_2_O_2_, *dps* expression was relatively low throughout the growth curve (Fig 2A). The addition of hydrogen peroxide induced *dps* differentially based on growth phase (Fig 2A), confirming our RNA-seq data that *dps* transcription was significantly induced by H_2_O_2_. *dps* induction by H_2_O_2_ was increased dramatically during exponential growth, similar to that in *E*. *coli* [44]. In stationary phase cultures, however, *dps* expression was induced less prominently (Fig 2A).

It has been reported that *dps* expression requires the redox sensor, OxyR in *E*. *coli*. [44]. To test whether *dps* is also regulated by OxyR in *V*. *cholerae*, we examined *dps* expression in *oxyR* deletion mutants. During exponential growth, compared to wild type, *dps* induction as a result of H_2_O_2_ exposure was abolished in *ΔoxyR* (Fig 2B, left panel). Complementation of *oxyR* on a plasmid restores *dps* induction when exposed to H_2_O_2_ (Fig 2B, left panel). These data suggest that OxyR induces *dps* expression upon exposure to hydrogen peroxide in exponential growth phases in *V*. *cholerae*, similar to that in *E*. *coli*. Upon entry into stationary phase, however, *dps* expression in *ΔoxyR* mutants was similar to that of wild type (Fig 2B, right panel), indicating that OxyR does not regulate *dps* at this growth phase. It has been reported that in *E*. *coli*, the major stationary phase regulator RpoS (σ^38^) is required for *dps* expression in stationary phase [44]. To test whether RpoS also regulates *dps* in *V*. *cholerae*, we compared *dps* expression between wild type and an *rpoS* in-frame deletion mutant. We found that in mid-log phase, RpoS did not affect *dps* expression (Fig 2B, left panel), whereas in stationary phase, *dps* expression was decreased in *ΔrpoS* mutants (Fig 2B, right panel). These data suggest that RpoS is the key regulator for *dps* in stationary phase.

### Dps is critical for *V*. *cholerae* resistance of inorganic and organic hydroperoxides

**Fig 3:**
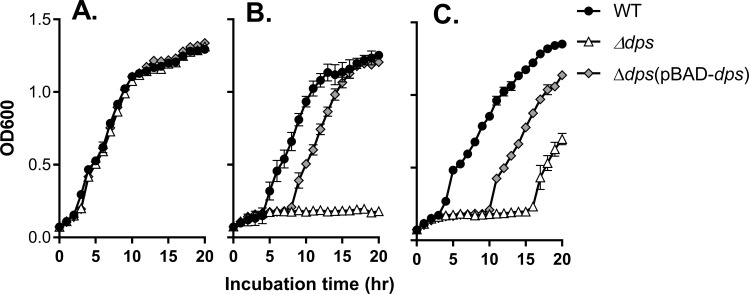
The effects of Dps and ROS on *V*. *cholerae* growth. Overnight cultures of wild type, *Δdps*, Δ*dps*(pBAD-*dps*) were diluted into LB containing 0.1% arabinose (**A**), with 250 μM H_2_O_2_ (**B**), or with 80 μM CHP (**C**). At the indicated time point, OD_600_ was measured. Results are means and s.d. of three independent experiments.

To investigate the role of Dps in *V*. *cholerae* ROS resistance, we constructed a *dps* in-frame deletion mutant to test the effect of *dps* and ROS on growth. We found that the growth of Δ*dps* was comparable to that of wild type (Fig 3A). However, in the presence of H_2_O_2_, Δ*dps* showed significantly reduced growth (Fig 3B, triangles). Expression of *dps in trans* largely restored the growth of Δ*dps* to wild type levels after 2 hours (Fig 3B, diamonds). Similarly, in the presence of organic hydroperoxide such as cumene hydroperoxide (CHP), Δ*dps* displayed significantly delayed growth compared to wild type cells (Fig 3C, triangles) and this defect was partially compensated when *dps* was expressed *in trans* (Fig 3C, diamonds). Taken together, these results suggest that Dps is important for *V*. *cholerae* growth in the presence of both organic and inorganic hydroperoxides.

### Dps is involved in resistance to multiple environmental stresses

**Fig 4:**
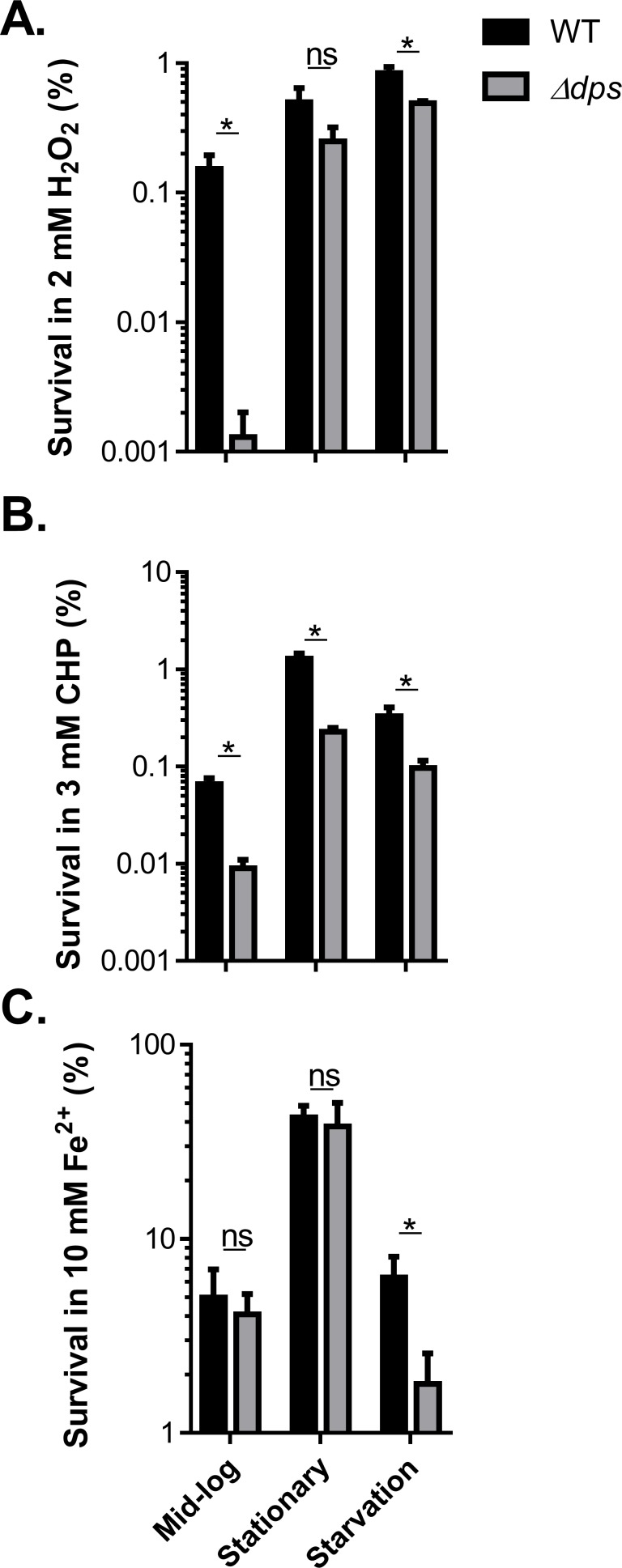
The effect of Dps on *V*. *cholerae* stress resistance. Wild type and Δ*dps* were grown in LB to mid-log and stationary phases. To induce starvation, wildtype and Δ*dps* were grown in LB until mid-log phase. The cells were then resuspended in ASW and incubated at 22°C for 2 days. A set of cultures was then exposed to 2 mM H_2_O_2_ (**A**), 3 mM CHP (**B**), or 10 mM FeSO_4_ (**C**) for 15 mins. Viable cells were then enumerated by serial dilution and subsequent plating on LB plates. Results are means and s.d. of three independent experiments. *: P<0.05; ns: no significance.

In addition to the well-studied role Dps plays in resistance to oxidative stress [24–26], it has also been shown to be important for resistance to other stresses, such as starvation [18, 45], osmotic stress [46], iron toxicity [25, 26, 28], and acid stress [27] in many bacteria. To test whether Dps is important for resistance to these stresses in *V*. *cholerae*, we compared the survival rate of wild type and Δ*dps* when exposed to different stress signals at different growth stages: exponential phase, stationary phase, and starvation. We found that *Δdps* mutants were more susceptible to H_2_O_2_ during exponential growth, but not at stationary phase (Fig 4A). The viability of Δ*dps* mutants under the starvation condition was similar to that of wild type (data not shown). However, upon exposure to H_2_O_2_, the number of *dps* mutants was significantly reduced (Fig 4A), suggesting that in starved cells Dps is critical for protecting *V*. *cholerae* against ROS. When cultures were exposed to organic hydroperoxide CHP, *Δdps* mutant cells were more susceptible than wild type cells at all tested growth phases (Fig 4B). We also exposed wild type and Δ*dps* mutants to high osmolality, acid shock, and high concentrations of iron. We found that Δ*dps* showed lower viability when exposed to high iron concentrations during starvation (Fig 4C). However, Δ*dps* displayed similar survival rates when exposed to high osmolality and low pH (data not shown). These data suggest that Dps is important for *V*. *cholerae* survival in starved cells as a response to ROS and for tolerating iron toxicity.

**Fig 5:**
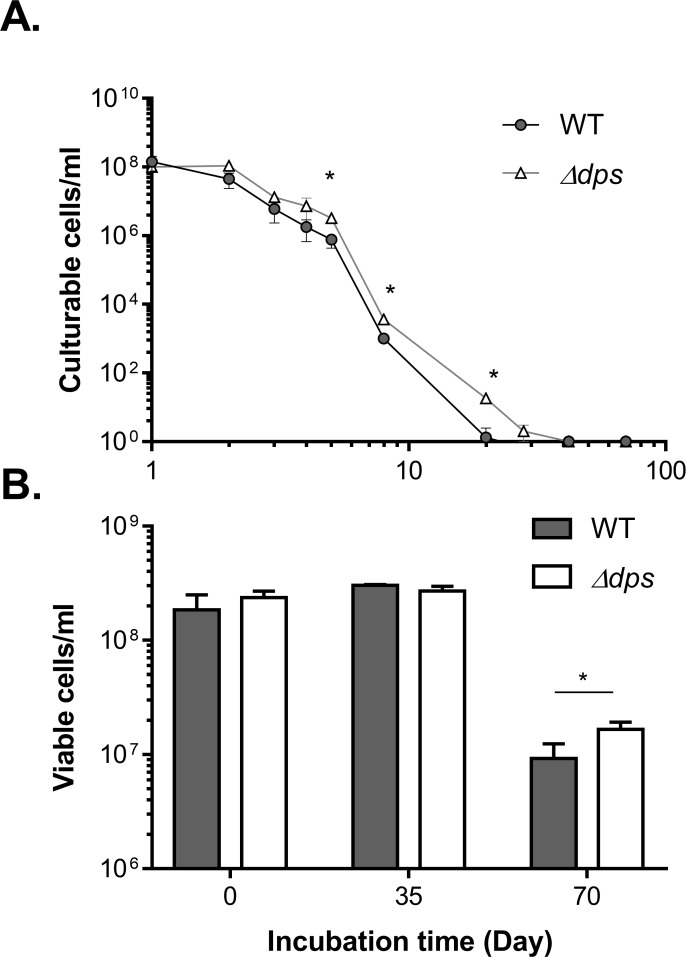
The effect of Dps on *V*. *cholerae* survival in the VBNC state. Mid-log cultures of wild type and Δ*dps* were washed and diluted in ASW and then incubated at 4°C for 70 days. At the time indicated, the samples were withdrawn and the number of culturable cells (**A**) was determined by plating the cell suspension on tryptic soy agar plates supplemented with 0.1% sodium pyruvate. The number of viable cells (**B**) was determined by using a real-time PCR method described in [38]. Results are means and s.d. of three independent experiments. *: P<0.05.

To examine additional roles of Dps with respect to *V*. *cholerae* stress resistance, we induced the production of “viable but non culturable (VBNC)” cells. It has been reported that upon exposure to unfavorable environments, *V*. *cholerae* can survive by entering a VBNC state [47], in which bacteria fail to grow on routine bacteriological media, but are alive and capable of resuscitation under favorable conditions such as *in vivo*. To test whether Dps is involved in *V*. *cholerae* survival in VBNC, we inoculated mid-log wild type and Δ*dps* into artificial sea water and incubated at 4°C. Culturable cells were determined by plating cell suspensions on rich medium agar plates, while viable cells were determined by using the PCR method described in [38]. Fig 5A shows that the percentage of culturable wild type cells declined rapidly, while a statistically significantly more culturable Δ*dps* cells were detected. Similarly, we found that there were more viable Δ*dps* cells than wild type cells after 70 days of incubation in ASW at 4°C (Fig 5B). These results suggest that Dps has a negative effect on cell viability under the VBNC condition tested. Interestingly, a previous transcriptome study shows that in the VBNC condition, *dps* expression is over 2-fold higher than that in vegetative cells [48]. This may imply that Dps is involved with the adaptation of cells to stresses such as cold temperatures and poor nutrients.

### Dps is important for *V*. *cholerae* colonization of inflammatory intestines

**Fig 6:**
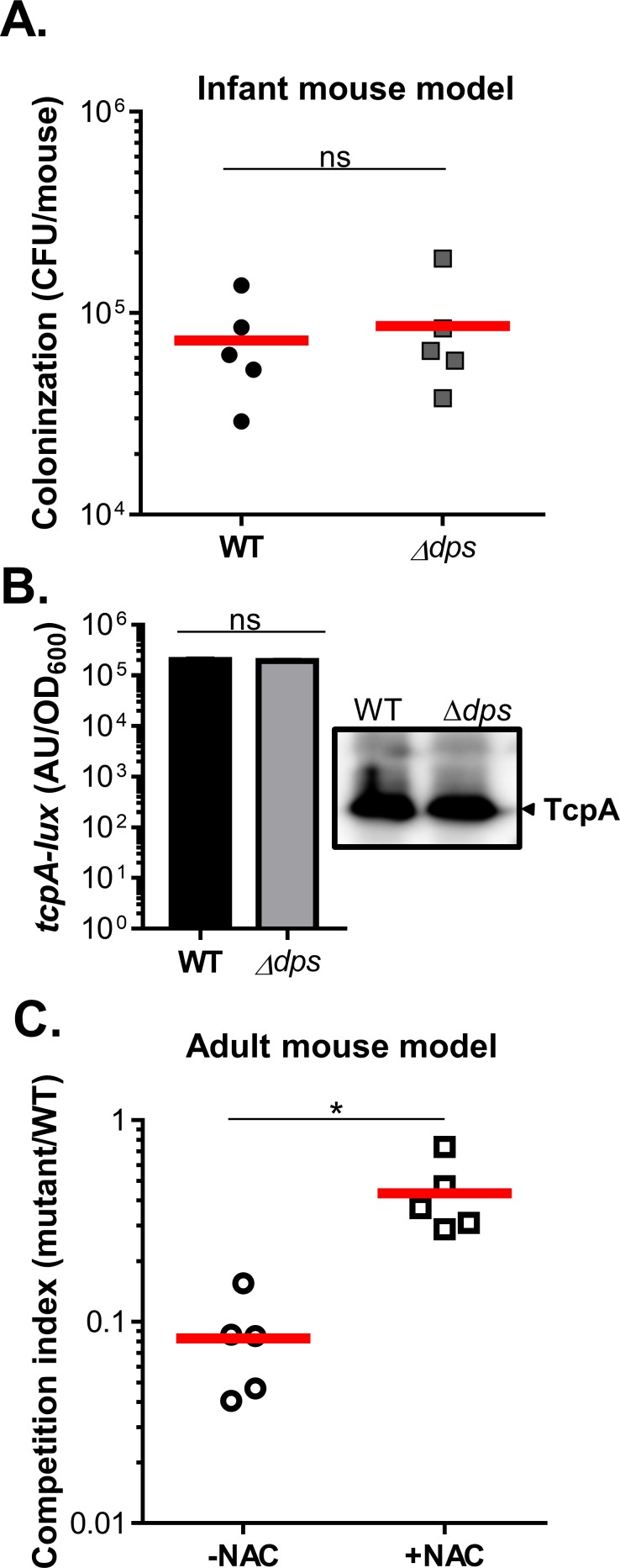
The effect of Dps on *V*. *cholerae* pathogenesis. **A.** Infant mouse colonization: Approximately 10^5^ wild type (*lacZ*^*-*^) and *dps* mutants (*lacZ*^*+*^) were intragastrically inoculated into 5-day-old CD-1 mice in a 1:1 ratio. After 18-hr incubation, CFU from small intestines were determined by serial dilution and plated on LB agar. The data shown are from three independent experiments and each symbol represents CFU recovered from one mouse intestine. Horizontal lines represent the average number of cells recovered. **B.** Virulence factor expression and production: wild type and Δ*dps* containing P_*tcpA*_-*luxCDABE* were grown under the virulence inducing AKI condition [37]. Luminescence was measured and normalized to OD_600_ (left panel), and 10^9^ cells were subjected to sodium dodecyl sulfate-polyacrylamide gel electrophoresis (SDS-PAGE) and immunoblotting using anti-TcpA antiserum (right panel). **C.** Colonization in adult mice: Five-week-old CD-1 mice were provided with drinking water with or without the antioxidant NAC for one week. Mice were then treated with streptomycin and intragastrically administered a 1:1 mixture of wild type (*lacZ*^*-*^) and Δ*dps* (*lacZ*^*+*^*)*. Fecal pellets were collected from each mouse at the indicated time points, resuspended in PBS, serially diluted, and then plated on plates containing X-gal. The competitive index (CI) was calculated as the ratio of mutant to wild type colonies normalized to the input ratio. Horizontal lines represent the average CI. *: P<0.05.

To investigate whether Dps plays a role in *V*. *cholerae* pathogenesis, we first used an infant mouse colonization model [49] to test for *dps* mutant colonization. We found that *dps* mutants could colonize in the small intestine of 5-day-old infant mice as well as wild type (Fig 6A). We also examined whether Dps affects the expression and production of TcpA, the major virulence factor in *V*. *cholerae* [50]. *In vitro tcpA* induction (Fig 6B, left panel) and TcpA production (Fig 6B, right panel), were similar between wild type and Δ*dps*, suggesting that Dps does not affect virulence gene expression. To examine the role of Dps in ROS resistance during infection, we performed an *in vivo* colonization competition assay using the streptomycin-treated adult mouse model in which bacteria experience host-generated oxidative and nitrosative stress [42, 51]. Fig 6C shows that Δ*dps* was outcompeted by wild type in this model. However, treatment with N-acetyl cysteine (NAC), an antioxidant widely used in human and animal studies to lower ROS levels [52], restores Δ*dps* colonization (Fig 6C, squares). These results suggest that Dps is important for ROS resistance *in vivo* [53, 54].

In this study, we show that similar to *E*. *coli* and other bacteria, the expression of *dps* is activated by OxyR and H_2_O_2_ during exponential growth in *V*. *cholerae*. At stationary phase, RpoS is important for *dps* expression. Like in other bacteria, Dps is critical for *V*. *cholerae* resistance to both inorganic and organic hydroperoxides as well as resistance to iron toxicity during specific growth phases. Interestingly, Dps also has an effect on the production of VBNCs, which may be important for V. cholerae as they reside in aquatic environments between infections. In addition, Dps plays a role in *V*. *cholerae* colonization and is critical for *V*. *cholerae in vivo* ROS resistance. Our study adds Dps as an additional factor to *V*. *cholerae*’s arsenal of tools used for survival in both aquatic and host environments. As Dps is well conserved in many bacteria, including pathogens, our study contributes to the knowledge of pathogenic mechanisms required to achieve successful infection.

## Supporting information

S1 Table Genes that differentially expressed more than 2-fold upon H_2_O_2_ exposure. Wild type *V*. *cholerae* were inoculated into virulence-inducing AKI medium and incubated at 37°C for 4 hrs. One set of cultures were then exposed to 0.5 mM H_2_O_2_ for 30 min. RNA was purified and RNA sequencing was performed by PrimBio Research Institute (Exton, PA, USA).
(DOCX)
