# Supplementary material for: OxyR-activated expression of Dps is important for Vibrio cholerae oxidative stress resistance and pathogenesis
Source: PLoS One. 2017 Feb 2;12(2):e0171201. doi: 10.1371/journal.pone.0171201 (PMC5289545; doi:10.1371/journal.pone.0171201)
Supplement: S1 Table — Wild type V. cholerae were inoculated into virulence-inducing AKI medium and incubated at 37°C for 4 hrs. One set of cultures were then exposed to 0.5 mM H2O2 for 30 min. RNA was purified and RNA sequencing was performed by PrimBio Research Institute (Exton, PA, USA). (DOCX) [file pone.0171201.s001.docx]

**Supplemental Materials**

**S1 Table. Genes that differentially expressed more than 2-fold upon H_2_O_2_ exposure.**

| **GeneID** | **function** | **-H_2_O_2_/+H_2_O_2_** | **p value** | **logFC** | **logCPM** | **LR** | **minFDR** |
| --- | --- | --- | --- | --- | --- | --- | --- |
| VC0091 | O-methyltransferase-related protein | -3.01 | 0.00 | -1.59 | 6.02 | 17.32 | 8.50 |
| VC0139 | DPS family protein | -27.39 | 0.00 | -4.78 | 9.47 | 55.89 | 34.30 |
| VC0199 | hemolysin secretion ATP-binding protein, putative | -2.46 | 0.03 | -1.30 | 4.62 | 11.19 | 5.15 |
| VC0200 | iron(III) compound receptor | -2.59 | 0.00 | -1.37 | 5.63 | 19.15 | 9.35 |
| VC0364 | bacterioferritin-associated ferredoxin | -5.02 | 0.00 | -2.33 | 7.78 | 34.86 | 19.92 |
| VC0365 | bacterioferritin | -2.48 | 0.01 | -1.31 | 5.06 | 15.89 | 7.63 |
| VC0384 | sulfite reductase (NADPH) flavoprotein alpha-component( EC:1.6.2.4 ) | -2.54 | 0.03 | -1.35 | 4.40 | 10.75 | 4.94 |
| VC0426 | hypothetical protein | -27.48 | 0.00 | -4.78 | 8.02 | 106.43 | 68.63 |
| VC0538 | thiosulfate ABC transporter, periplasmic thiosulfate-binding protein | -3.80 | 0.00 | -1.93 | 6.37 | 24.90 | 13.20 |
| VC0747 | hypothetical protein | -2.57 | 0.01 | -1.36 | 6.50 | 14.36 | 6.85 |
| VC0748 | cysteine desulfurase( EC:2.8.1.7 ) | -3.19 | 0.00 | -1.67 | 7.89 | 18.28 | 8.93 |
| VC0749 | NifU-related protein | -3.13 | 0.00 | -1.65 | 7.15 | 17.43 | 8.54 |
| VC0750 | hesB family protein | -3.54 | 0.00 | -1.82 | 6.65 | 18.84 | 9.19 |
| VC0788 | DOPA-dioxygenase-related protein | -13.41 | 0.00 | -3.75 | 6.53 | 81.97 | 51.80 |
| VC0968 | cysteine synthase A( EC:2.5.1.47 ) | -2.74 | 0.01 | -1.45 | 6.57 | 13.94 | 6.67 |
| VC0995 | PTS system, N-acetylglucosamine-specific IIABC component | -2.20 | 0.02 | -1.14 | 7.58 | 11.95 | 5.55 |
| VC1096 | hypothetical protein | -10.47 | 0.00 | -3.39 | 5.86 | 41.71 | 24.46 |
| VC1235 | sodium/dicarboxylate symporter | -2.43 | 0.03 | -1.28 | 4.58 | 11.23 | 5.15 |
| VC1560 | catalase/peroxidase( EC:1.11.1.6 ) | -7.37 | 0.00 | -2.88 | 7.28 | 52.53 | 32.25 |
| VC1585 | catalase( EC:1.11.1.6 ) | -3.18 | 0.05 | -1.67 | 3.59 | 9.79 | 4.38 |
| VC2111 | hypothetical protein | -15.58 | 0.00 | -3.96 | 6.89 | 60.27 | 36.93 |
| VC2211 | ferric vibriobactin receptor | -2.91 | 0.00 | -1.54 | 4.52 | 16.55 | 8.04 |
| VC2481 | D-3-phosphoglycerate dehydrogenase( EC:1.1.1.95 ) | -2.18 | 0.03 | -1.12 | 6.53 | 11.19 | 5.15 |
| VC2605 | hypothetical protein | -4.19 | 0.00 | -2.07 | 6.01 | 25.46 | 13.55 |
| VC2637 | peroxiredoxin family protein/glutaredoxin | -10.63 | 0.00 | -3.41 | 6.20 | 55.39 | 34.16 |
| VC2638 | dihydrolipoamide dehydrogenase( EC:1.8.1.4 ) | -10.19 | 0.00 | -3.35 | 6.25 | 58.82 | 36.19 |
| VC2694 | superoxide dismutase, Mn( EC:1.15.1.1 ) | -2.06 | 0.03 | -1.04 | 5.59 | 11.11 | 5.13 |
| VCA0242 | hexulose-6-phosphate synthase SgbH, putative | -2.37 | 0.04 | -1.25 | 7.57 | 10.41 | 4.73 |
| VCA0302 | hypothetical protein | -4.08 | 0.01 | -2.03 | 4.68 | 15.49 | 7.44 |
| VCA0428 | hypothetical protein | -2.38 | 0.03 | -1.25 | 5.12 | 10.80 | 4.96 |
| VCA0470 | acetyltransferase, putative | -2.17 | 0.01 | -1.12 | 5.44 | 13.79 | 6.57 |
| VCA0907 | hypothetical protein | -2.43 | 0.05 | -1.28 | 6.43 | 9.90 | 4.44 |
| VCA0909 | coproporphyrinogen III oxidase | -5.41 | 0.00 | -2.44 | 5.20 | 38.32 | 22.37 |
| VCA0963 | hypothetical protein | -2.59 | 0.02 | -1.37 | 4.57 | 11.65 | 5.41 |
| VCA0969 | pirin-related protein | -13.71 | 0.00 | -3.78 | 6.61 | 63.27 | 38.72 |
| VCA1087 | anti-sigma F factor antagonist, putative | -2.60 | 0.05 | -1.38 | 5.54 | 9.74 | 4.35 |
| VC0034 | thiol:disulfide interchange protein | 2.09 | 0.01 | 1.07 | 6.28 | 13.22 | 6.20 |
| VC0218 | ribosomal protein L28 | 3.47 | 0.01 | 1.79 | 9.74 | 15.38 | 7.43 |
| VC0219 | 50S ribosomal protein L33 | 2.96 | 0.05 | 1.57 | 9.86 | 9.85 | 4.42 |
| VC0273 | DNA-binding protein HU | 4.08 | 0.00 | 2.03 | 9.63 | 16.75 | 8.14 |
| VC0290 | DNA-binding protein Fis | 2.39 | 0.05 | 1.26 | 5.74 | 9.91 | 4.44 |
| VC0306 | thioredoxin | 3.39 | 0.01 | 1.76 | 9.78 | 14.16 | 6.78 |
| VC0307 | transcription termination factor Rho | 2.83 | 0.00 | 1.50 | 8.04 | 17.56 | 8.57 |
| VC0322 | translocase | 2.22 | 0.05 | 1.15 | 8.31 | 9.72 | 4.35 |
| VC0354 | peptidyl-prolyl cis-trans isomerase, FKBP-type | 4.04 | 0.01 | 2.01 | 9.49 | 15.67 | 7.55 |
| VC0397 | single-strand DNA-binding protein | 2.98 | 0.01 | 1.58 | 8.88 | 15.46 | 7.44 |
| VC0424 | hypothetical protein | 2.27 | 0.02 | 1.18 | 5.69 | 12.32 | 5.73 |
| VC0451 | hypothetical protein | 3.31 | 0.00 | 1.73 | 7.09 | 20.85 | 10.41 |
| VC0483 | hypothetical protein | 2.34 | 0.04 | 1.23 | 8.30 | 10.50 | 4.78 |
| VC0519 | hypothetical protein | 3.14 | 0.02 | 1.65 | 9.71 | 11.52 | 5.32 |
| VC0520 | 30S ribosomal protein S21 | 3.67 | 0.02 | 1.88 | 9.55 | 12.68 | 5.89 |
| VC0596 | dnaK suppressor protein | 2.86 | 0.00 | 1.51 | 7.01 | 18.82 | 9.19 |
| VC0620 | peptide ABC transporter, periplasmic peptide-binding protein | 2.64 | 0.01 | 1.40 | 6.72 | 14.13 | 6.78 |
| VC0687 | carbon starvation protein A, putative | 3.69 | 0.00 | 1.88 | 7.24 | 25.91 | 13.81 |
| VC0790 | transcriptional regulator CitB | 2.04 | 0.03 | 1.03 | 5.11 | 11.21 | 5.15 |
| VC0798 | citrate lyase, beta subunit( EC:4.1.3.34 ) | 2.75 | 0.04 | 1.46 | 7.98 | 10.36 | 4.71 |
| VC0910 | PTS system, trehalose-specific IIBC component | 3.74 | 0.00 | 1.90 | 5.87 | 28.55 | 15.62 |
| VC0965 | phosphoenolpyruvate-protein phosphotransferase( EC:2.7.3.9 ) | 2.48 | 0.02 | 1.31 | 7.30 | 12.23 | 5.70 |
| VC0966 | phosphocarrier protein HPr | 4.63 | 0.00 | 2.21 | 10.06 | 18.08 | 8.92 |
| VC0984 | cholera toxin transcriptional activator | 2.10 | 0.05 | 1.07 | 5.84 | 9.94 | 4.44 |
| VC1077 | hypothetical protein | 5.09 | 0.00 | 2.35 | 7.57 | 41.08 | 24.15 |
| VC1115 | dithiobiotin synthetase( EC:6.3.3.3 ) | 2.68 | 0.01 | 1.42 | 8.47 | 15.71 | 7.55 |
| VC1154 | hypothetical protein | 2.36 | 0.04 | 1.24 | 7.82 | 10.35 | 4.71 |
| VC1178 | hypothetical protein | 2.23 | 0.02 | 1.16 | 6.49 | 11.79 | 5.46 |
| VC1182 | thioredoxin reductase( EC:1.8.1.9 ) | 2.14 | 0.02 | 1.10 | 5.85 | 12.00 | 5.56 |
| VC1189 | hypothetical protein | 2.24 | 0.02 | 1.16 | 6.28 | 12.57 | 5.82 |
| VC1249 | hypothetical protein | 2.31 | 0.02 | 1.21 | 7.11 | 11.70 | 5.43 |
| VC1301 | serine transporter | 5.43 | 0.00 | 2.44 | 7.39 | 29.02 | 15.89 |
| VC1343 | peptidase, M20A family | 3.80 | 0.00 | 1.93 | 6.62 | 31.78 | 17.84 |
| VC1423 | hypothetical protein | 2.40 | 0.01 | 1.26 | 6.74 | 13.21 | 6.20 |
| VC1428 | putrescine/spermidine ABC transporter ATPase( EC:3.6.3.30 ) | 2.64 | 0.00 | 1.40 | 5.44 | 17.49 | 8.55 |
| VC1491 | dihydroorotate dehydrogenase( EC:1.3.3.1 ) | 2.57 | 0.00 | 1.36 | 5.54 | 16.75 | 8.14 |
| VC1507 | 3-deoxy-7-phosphoheptulonate synthase( EC:2.5.1.54 ) | 2.01 | 0.04 | 1.01 | 4.99 | 10.11 | 4.55 |
| VC1508 | hypothetical protein | 2.68 | 0.01 | 1.42 | 5.78 | 15.77 | 7.57 |
| VC1531 | hypothetical protein | 5.69 | 0.00 | 2.51 | 5.40 | 18.19 | 8.93 |
| VC1702 | hypothetical protein | 3.38 | 0.01 | 1.76 | 6.69 | 15.26 | 7.37 |
| VC1722 | hypothetical protein | 2.45 | 0.05 | 1.29 | 7.00 | 9.72 | 4.35 |
| VC1737 | translation initiation factor IF-1 | 2.88 | 0.04 | 1.53 | 5.76 | 10.57 | 4.82 |
| VC1821 | PTS system, fructose-specific IIBC component | 4.21 | 0.00 | 2.07 | 6.92 | 20.16 | 9.95 |
| VC1826 | PTS system, fructose-specific IIABC component | 3.23 | 0.02 | 1.69 | 7.84 | 12.54 | 5.82 |
| VC1897 | Hit family protein | 2.44 | 0.00 | 1.29 | 6.46 | 16.17 | 7.81 |
| VC1918 | peptidyl-prolyl cis-trans isomerse D | 2.56 | 0.00 | 1.35 | 6.57 | 16.22 | 7.82 |
| VC1919 | DNA-binding protein HU-beta | 4.65 | 0.02 | 2.22 | 10.79 | 12.80 | 5.95 |
| VC1923 | trigger factor | 3.24 | 0.00 | 1.70 | 8.56 | 19.54 | 9.59 |
| VC1957 | hypothetical protein | 3.05 | 0.01 | 1.61 | 6.68 | 13.16 | 6.19 |
| VC1963 | hypothetical protein | 2.56 | 0.01 | 1.35 | 5.82 | 14.01 | 6.70 |
| VC2044 | hypothetical protein | 2.60 | 0.02 | 1.38 | 8.22 | 11.88 | 5.51 |
| VC2096 | seqA protein | 2.72 | 0.00 | 1.44 | 6.26 | 18.76 | 9.19 |
| VC2099 | flavodoxin | 2.39 | 0.01 | 1.26 | 6.97 | 14.81 | 7.09 |
| VC2156 | lipoprotein-34 NlpB | 2.31 | 0.02 | 1.21 | 7.02 | 12.43 | 5.77 |
| VC2159 | glycine cleavage system transcriptional repressor, putative | 2.07 | 0.04 | 1.05 | 6.27 | 10.30 | 4.68 |
| VC2174 | UDP-sugar hydrolase( EC:3.1.3.5 ) | 2.57 | 0.01 | 1.36 | 7.34 | 13.22 | 6.20 |
| VC2184 | peptidyl-tRNA hydrolase( EC:3.1.1.29 ) | 2.11 | 0.02 | 1.08 | 5.91 | 11.69 | 5.43 |
| VC2225 | uracil phosphoribosyltransferase | 2.21 | 0.04 | 1.15 | 6.54 | 10.08 | 4.53 |
| VC2239 | nitrogen regulatory protein P-II | 2.46 | 0.03 | 1.30 | 7.26 | 11.12 | 5.13 |
| VC2257 | ribosome releasing factor | 2.97 | 0.01 | 1.57 | 8.08 | 14.96 | 7.16 |
| VC2259 | elongation factor Ts | 2.65 | 0.03 | 1.40 | 8.78 | 11.28 | 5.16 |
| VC2267 | transcription antitermination protein NusB | 2.82 | 0.02 | 1.50 | 7.94 | 12.35 | 5.73 |
| VC2305 | outer membrane protein OmpK | 4.45 | 0.00 | 2.15 | 7.95 | 32.81 | 18.51 |
| VC2326 | hypothetical protein | 2.18 | 0.03 | 1.12 | 6.67 | 11.10 | 5.13 |
| VC2352 | NupC family protein | 3.59 | 0.00 | 1.84 | 9.33 | 18.20 | 8.93 |
| VC2441 | hypothetical protein | 2.38 | 0.01 | 1.25 | 7.04 | 14.22 | 6.78 |
| VC2448 | CTP synthetase( EC:6.3.4.2 ) | 2.70 | 0.02 | 1.43 | 8.94 | 12.33 | 5.73 |
| VC2512 | hypothetical protein | 2.73 | 0.01 | 1.45 | 8.27 | 14.29 | 6.81 |
| VC2544 | fructose-1,6-bisphosphatase( EC:3.1.3.11 ) | 2.66 | 0.00 | 1.41 | 6.92 | 18.11 | 8.92 |
| VC2614 | cyclic AMP receptor protein | 4.37 | 0.01 | 2.13 | 10.22 | 14.66 | 7.02 |
| VC2653 | export protein SecB | 3.65 | 0.00 | 1.87 | 8.88 | 20.85 | 10.41 |
| VC2660 | elongation factor P | 2.72 | 0.01 | 1.44 | 8.29 | 15.39 | 7.43 |
| VC2670 | triosephosphate isomerase( EC:5.3.1.1 ) | 2.93 | 0.02 | 1.55 | 8.24 | 12.78 | 5.95 |
| VC2699 | anaerobic C4-dicarboxylate transporter | 3.38 | 0.01 | 1.76 | 7.70 | 14.75 | 7.07 |
| VC2709 | DNA-directed RNA polymerase omega subunit | 3.91 | 0.00 | 1.97 | 7.89 | 22.95 | 11.80 |
| VCA0035 | phosphatidylglycerophosphatase B, putative | 2.09 | 0.05 | 1.06 | 5.86 | 9.86 | 4.42 |
| VCA0079 | hypothetical protein | 2.25 | 0.04 | 1.17 | 6.64 | 10.58 | 4.82 |
| VCA0125 | hypothetical protein | 2.46 | 0.02 | 1.30 | 6.68 | 11.97 | 5.56 |
| VCA0139 | hypothetical protein | 2.67 | 0.02 | 1.42 | 6.99 | 12.44 | 5.77 |
| VCA0184 | cold shock DNA-binding domain protein | 5.16 | 0.02 | 2.37 | 10.40 | 12.00 | 5.56 |
| VCA0205 | anaerobic C4-dicarboxylate transporter | 2.66 | 0.03 | 1.41 | 8.40 | 11.02 | 5.08 |
| VCA0277 | glycine cleavage system protein H | 4.28 | 0.00 | 2.10 | 7.57 | 19.78 | 9.72 |
| VCA0278 | serine hydroxymethyltransferase( EC:2.1.2.1 ) | 2.06 | 0.03 | 1.04 | 6.54 | 11.41 | 5.26 |
| VCA0478 | hypothetical protein | 2.12 | 0.05 | 1.08 | 7.00 | 9.92 | 4.44 |
| VCA0547 | hypothetical protein | 6.32 | 0.02 | 2.66 | 12.13 | 11.75 | 5.45 |
| VCA0573 | DamX-related protein | 3.17 | 0.00 | 1.66 | 7.00 | 22.13 | 11.26 |
| VCA0607 | nucleoside diphosphate kinase regulator | 2.55 | 0.02 | 1.35 | 6.29 | 12.85 | 5.96 |
| VCA0721 | hypothetical protein | 3.19 | 0.00 | 1.67 | 7.21 | 16.72 | 8.14 |
| VCA0741 | hypothetical protein | 5.51 | 0.01 | 2.46 | 10.58 | 14.92 | 7.15 |
| VCA0743 | hypothetical protein | 2.58 | 0.02 | 1.37 | 7.76 | 12.09 | 5.60 |
| VCA0744 | glycerol kinase( EC:2.7.1.30 ) | 2.45 | 0.04 | 1.29 | 8.29 | 10.46 | 4.76 |
| VCA0773 | methyl-accepting chemotaxis protein | 2.19 | 0.01 | 1.13 | 5.82 | 13.49 | 6.36 |
| VCA0774 | UDP-glucose 4-epimerase( EC:5.1.3.2 ) | 2.67 | 0.00 | 1.42 | 7.17 | 18.60 | 9.11 |
| VCA0784 | hypothetical protein | 2.30 | 0.02 | 1.20 | 7.02 | 12.26 | 5.70 |
| VCA0806 | hypothetical protein | 3.55 | 0.01 | 1.83 | 7.25 | 14.15 | 6.78 |
| VCA0893 | hypothetical protein | 3.89 | 0.00 | 1.96 | 5.93 | 26.71 | 14.33 |
| VCA0933 | cold shock domain family protein | 6.15 | 0.00 | 2.62 | 8.30 | 40.27 | 23.68 |
| VCA1063 | ornithine decarboxylase, inducible( EC:4.1.1.17 ) | 2.49 | 0.01 | 1.32 | 5.65 | 14.43 | 6.87 |
| VCA1079 | pyridoxamine 5'-phosphate oxidase( EC:1.4.3.5 ) | 2.05 | 0.03 | 1.04 | 6.69 | 10.92 | 5.02 |

GeneID= locus tag,  logCPM=log2 Counts-Per-Million, LR=Likelihood Ratio, p-value=t-test pvalue,  minFDR=-log2(adj-pvalue).
